# Choice of geographic unit influences socioeconomic inequalities in breast cancer survival

**DOI:** 10.1038/sj.bjc.6602506

**Published:** 2005-03-29

**Authors:** L M Woods, B Rachet, M P Coleman

**Affiliations:** 1 Non-communicable Disease Epidemiology Unit, London School of Hygiene and Tropical Medicine, Keppel Street, London WC1E 7HT, UK

**Keywords:** deprivation, ecological studies, small-area geography, socioeconomic status, survival

## Abstract

Socioeconomic differences in age-standardised crude survival for women diagnosed with breast cancer during 1991–1999 in England were influenced by the population of the geographic area used to assign the deprivation index, but not by the choice of index.

In the absence of individual data on personal circumstances, the socioeconomic status of cancer patients has often been determined using a census-derived or area-based score designed to reflect some aspect of material deprivation or socioeconomic status of the small geographic area in which a person resides. Using such an ecologic approach, large differences in cancer patient survival have been described in various countries (Boyd *et al*, 1999; Coleman *et al*, 2001, 2004; Bradley *et al*, 2001). However, it is not known to what extent the observed variability in the deprivation gradients is due to the nature of the small-area geographies in question, the years to which the deprivation data apply, or the particular deprivation index used. This analysis aims to clarify the impact of each of these factors with specific reference to breast cancer survival in England in the 1990s. It was originally conducted in order to determine the most suitable deprivation geography to use in a large-scale cancer survival study in which a single index of deprivation could not be consistently applied across the study period (Coleman *et al*, 2004).

## MATERIALS AND METHODS

Table 1 summarises the deprivation measures applied. We used three deprivation indices for the period 1991–2001: the census-derived Townsend score (Townsend *et al*, 1988) and the Indices of Multiple Deprivation (IMD) 2000 (Department of the Environment, 2000) and 2004 (Neighbourhood Renewal Unit, 2004) comprising a number of indices of deprivation for different domains of life. Each IMD domain is based upon routinely available administrative data. Indices were calculated using several different definitions of small geographic area as the basis of analysis, consisting of census Enumeration District (ED) 1991, electoral ward 1991 and 1998, census-defined Standard Table wards 2001 and Lower-Level Super Output Area 2001 (SOA, a new census geography). Table 2 summarises the number of geographic units in England, and the mean, standard deviation (s.d.) and coefficient of variation (CV) of their population. The Townsend score was calculated for two geographic levels in 1991 and 2001. The IMD 2000 was available from data applying to 1998, for each electoral ward as defined in 1998, while the IMD 2004 used data from 2000 and 2001 for each SOA as defined in 2001. We used only the income, employment and education domains of the IMD 2000 and 2004, since these domains displayed the greatest temporal consistency of definition between 1998 and 2001. We excluded the health domain because it was likely to be autocorrelated with cancer survival. We also examined a crude measure of material deprivation: the proportion of adults registered as being in receipt of income support during 1995 for each of the electoral wards defined in 1998. In all, deprivation was categorised by 11 unique combinations of geographic unit, data time point and deprivation index. Each set of small areas in England was ranked on the relevant index and divided into five deprivation categories defined by quintiles of its national distribution, numbered from 1 (the most affluent areas) to 5 (the most deprived areas).

All women resident in England diagnosed aged 15 or over with malignant breast cancer during the period 1 January 1991 to 31 December 1999 were eligible for inclusion. Tumour records were excluded if they represented a bilateral, synchronous or second tumour (4.2%), if survival time or vital status was unknown (6.6%), if the woman was aged 100 years or over at diagnosis (0.1%) or if baseline data fields were inconsistent (<0.1%). Follow-up was complete on all women to 31 December 2001. A deprivation category for each of the 11 unique combinations was assigned to each individual patient on the basis of her postcode at diagnosis. One percent of records could not be linked to one or more of the 11 combinations due to missing geographic information, and these were excluded from all analyses. A total of 246 611 women (88% of those eligible) were included in the analyses.

Crude survival was estimated using individual survival times. Five-year survival rates were directly age standardised using a detailed set of 12 age groups (15–34, 35–39, …, 80–84 and 85–99) in order to adjust as fully as possible for any differences between deprivation categories in the age structure of women with breast cancer. The age structure of all the women included in the analyses was used as the standard.

The deprivation ‘gap’ in survival between the most affluent and the most deprived categories was estimated using least-squares linear regression. As indicated in Table 1, 11 different models were fitted, one for each unique combination of geographic unit, data time point and deprivation index. Each model was fitted upon the five quintile-specific age-standardised survival rates, each weighted by its standard error. A nonlinear relationship between deprivation and survival was also modelled using a quadratic term (*y*=*a*+*bx*+*cx*^2^). The statistical significance of differences in the survival gradient between any two models was assessed by fitting a single model to the 10 quintile-specific rates in question, with and without an interaction term, and applying the likelihood ratio test. All results were evaluated at the 95% significance level.

## RESULTS

A significant quadratic component was found in the association between crude survival and socioeconomic deprivation with all 11 combinations of geographic unit, data time point and deprivation index.

### Impact of geography and time point

**Figure 1:**
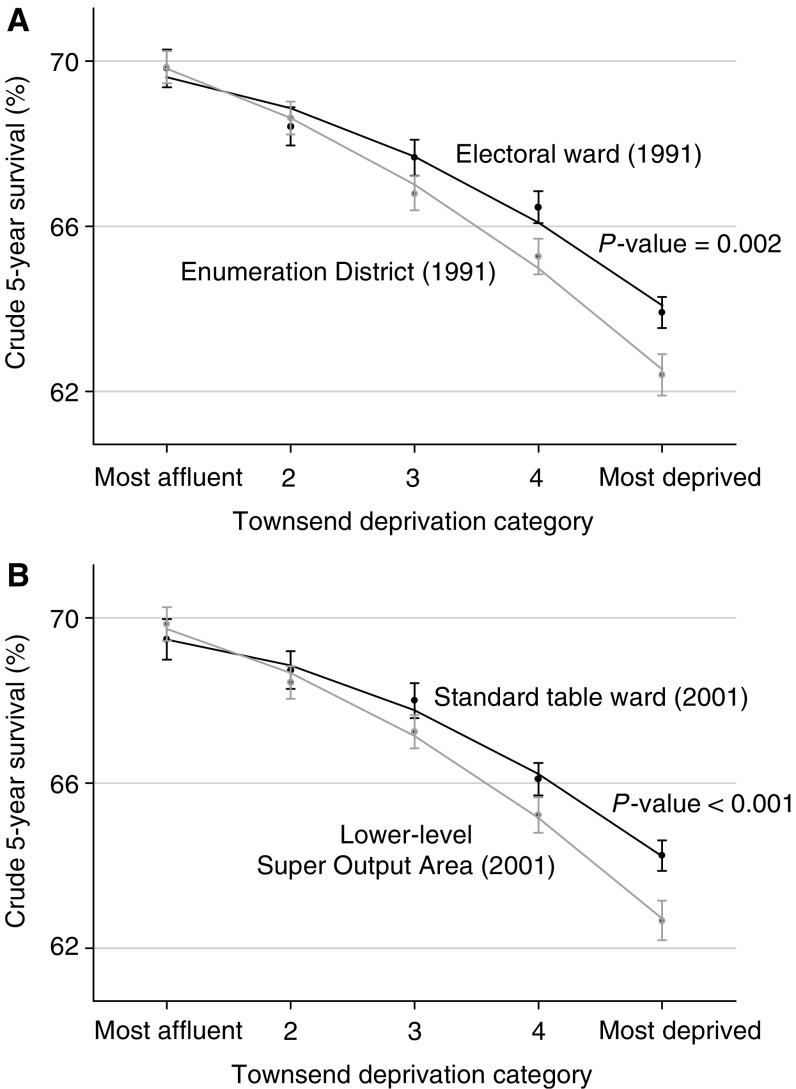
Crude age-standardised 5-year survival, with 95% confidence intervals, by Townsend deprivation category and associated fitted quadratic regression lines in (**A**) 1991 and (**B**) 2001: England, women (15–99 years) diagnosed with breast cancer 1991–1999 and followed up until 2001. *P*-values originate from a likelihood ratio test of the interaction between deprivation category and geographic unit of analysis (see text).

The deprivation gap in 5-year survival based on the 1991 Townsend score for electoral wards (−5.53%) was significantly smaller than that based on EDs (−7.28%), for which the average population is less than a tenth that of wards (Table 2, Figure 1A). Similarly, the deprivation gap in survival based on Townsend scores for the 2001 Standard Table wards (−5.25%) was significantly smaller than that estimated with SOA-based Townsend scores (−7.01%). SOAs are about one-third the size of wards (Table 2, Figure 1B). In contrast, there was no significant difference between the deprivation gaps in survival assessed with Townsend scores based on wards for 1991 and 2001, for which the average populations were very similar. In addition, no significant difference was observed between the deprivation survival gap based upon the Townsend score for EDs in 1991 and SOAs in 2001, despite the fact that SOAs are three times larger than EDs.

### Impact of deprivation score

For a given geographic unit of analysis and the same data time point, the deprivation gap in survival was similar for all measures of deprivation (Table 2). The deprivation gaps for income support 1995 and the income domain of IMD 2000 were not significantly different, nor was there a significant difference between the gaps identified by the income and employment domains of the IMD 2000. The IMD 2000 education domain identified a slightly but significantly smaller gap (−5.28%) than both the employment and income domains, but the differences were all less than 1%. The deprivation gap in survival varied between −7.01 and −7.44% with the four deprivation indices measured at SOA level in 2001, but none of these small differences was significant.

## DISCUSSION

We have shown that when using an ecologic approach, the measurement of deprivation differentials in breast cancer survival in England during the late 20th century was primarily influenced by the population of the geographic area for which the deprivation index is derived, rather than by the definition of the index itself.

Although small in absolute terms, the influence of underlying geography is striking. For a given time point and deprivation score, the deprivation gap in crude survival was some 25% smaller when estimated with large geographic units than with small ones. These differences represent a dilution effect caused by the larger population of the larger area, and the associated increase in social heterogeneity. For example, the mean population of EDs in 1991 was about 10 times smaller than that of the 1991 electoral wards, and had a much smaller CV (see Table 2). The differences in population size and homogeneity between SOAs and Standard Table wards in 2001 were also marked.

The interpretation of these patterns as a dilution effect is supported by three other observations. First, the impact of geography is seen primarily in the most deprived quintiles. The mean population size of both 1991 electoral wards and 2001 Standard Table wards increases with increasing deprivation, while for EDs and SOAs, the population size of each quintile is fairly constant (data not shown). These patterns result in the mean population of deprived wards being much larger than that of deprived EDs or SOAs, while the population size of affluent wards is more similar to that of affluent EDs and SOAs. This leads to greater attenuation in survival for the more deprived categories. Second, the survival gap estimated with ward-based Townsend scores was similar for the 1991 and 2001 wards, which had a similar mean population size and CV (Table 2). Finally, the deprivation gaps in survival identified for SOAs in 2001 and EDs in 1991 were not significantly different. In this case, although SOAs have a population three times the size of EDs in 1991, they are much more homogeneous with respect to their size (CV 13 *vs* 37% Table 2) and their social characteristics (Martin, 2002). It appears that the joint effect of these two competing factors is to produce a very similar deprivation gap in survival for EDs and SOAs.

The definition of the deprivation index had little impact on the survival gap. Thus for electoral wards in 1998, the estimated deprivation gap in survival was very similar when deprivation was categorised either by the proportion of the population on income support or by the more complex income domain score, suggesting that the simpler index was just as useful for detecting socioeconomic differences in cancer survival. However, the use of two or more different scores may help in understanding the meaning and/or the mechanisms of such inequalities. Categorisation of deprivation using educational rather than employment or income variables led to a smaller deprivation gap in survival using IMD 2000 (based on wards), but not IMD 2004 (based on SOAs). Since the constituent variables for IMD 2000 and IMD 2004 are similar, this may again indicate attenuation of the effect of deprivation on survival with the larger geographic unit.

This analysis has only examined survival from breast cancer among women, but is probable that these observations apply similarly to incidence or mortality data and other diseases in both sexes. Our results are consistent with some of the literature on this topic, which has shown that the choice of deprivation score has little impact upon socioeconomic differences in morbidity (Hoare, 2003), and that underlying geography is influential in mortality and cancer incidence differentials (Krieger *et al*, 2002). Our results are less consistent with the conclusions of three other published studies, in which differences in the explanatory power of several deprivation indices were detected (Morris and Carstairs, 1991) and no impact of small-area geography was observed (Carr-Hill and Rice, 1995; Reijneveld *et al*, 2000). This may be due to the fact that these studies used outcomes other than cancer (Morris and Carstairs, 1991), or because the populations of the geographic areas examined were much more similar (Reijneveld *et al*, 2000), or a result of the analytic approach applied in which individual variables were correlated with area-derived data (Carr-Hill and Rice, 1995). Our study adds a cancer-specific component to this literature, and demonstrates the primary importance of deprivation geography when interpreting ecologic studies on socioeconomic differences in cancer survival.

## Figures and Tables

**Table 1:** Geographic unit of analysis (with year of its definition) and deprivation index (with the data year): 11 unique combinations used in survival analysis

**Deprivation index (data year)**	**Geographic unit of analysis and year of its definition**				
	**1991**		**1998**	**2001**	
	**ED**	**Electoral ward**	**Electoral ward**	**Standard Table ward**	**Lower-level SOA**
Townsend score (1991 and 2001)	×	×		×	×

*IMD 2000 (1998)*					
Income domain			×		
Employment domain			×		
Education domain			×		

*IMD 2004 (2000/2001)*					
Income domain					×
Employment domain					×
Education domain					×

Income support claimants (1995)			×		

ED=Enumeration District; SOA=Super Output Area; IMD=Indices of Multiple Deprivation.

**Table 2:** Characteristics of geographic units of analysis and fitted deprivation gaps (%) in 5-year crude survival derived from quadratic regression, by geographic unit of analysis and deprivation index: England, women (15–99 years) diagnosed with breast cancer 1991–1999 and followed up until 2001

**Geographic unit of analysis (year of its definition)**	**No. of units (England)**	**Mean population**	**s.d.**	**CV (%)**	**Fitted deprivation gap (%)^a^**
*Deprivation index (data year)*					
Census ED (1991)	106 865	440	164	37	
Townsend score (1991)					−7.28
Electoral ward (1991)	8985	5237	4042	77	
Townsend score (1991)					−5.53
Electoral ward (1998)	8414	5883	4212	72	
Percent on income support (1995)					−5.79
IMD 2000 education (1998)					−5.28
IMD 2000 employment (1998)					−5.78
IMD 2000 income (1998)					−6.04
Lower-level SOA (2001)	32 482	1513	199	13	
Townsend score (2001)					−7.01
IMD 2004 education (2000/2001)					−7.07
IMD 2004 employment (2000/2001)					−7.34
IMD 2004 income (2000/2001)					−7.44
Standard Table ward (2001)	7932	6195	4108	66	
Townsend score (2001)					−5.25

s.d.=standard deviation; CV=coefficient of variation; ED=Enumeration District; SOA=Super Output Area; IMD=Indices of Multiple Deprivation.

a Absolute difference in fitted crude 5-year survival (%) between the most affluent and most deprived categories. A negative gap indicates that survival is worse in the most deprived category.
